# Paediatric uveal melanoma in Ireland 2010 – 2024: incidence, clinical features, management, and outcomes

**DOI:** 10.1038/s41433-026-04334-2

**Published:** 2026-02-25

**Authors:** Denis Nevrov, Matthew O’Riordan, Huda Fadlelseed, Fiona D’Arcy, Joaquina Martinez Vicente, Michael Capra, Patrick Murtagh, Susan Kennedy, Noel Horgan

**Affiliations:** 1 Royal Victoria Eye and Ear Hospital, Dublin, Ireland; 2 Research Foundation, Royal Victoria Eye and Ear Hospital, Dublin, Ireland; 3 National Ophthalmic Pathology Laboratory, Royal Victoria Eye and Ear Hospital, Dublin, Ireland; 4 Children’s Health Ireland at Crumlin, Dublin, Ireland; 5 UCD School of Medicine, University College Dublin, Dublin, Ireland

**Keywords:** Eye cancer, Eye cancer

## Abstract

**Background:**

Uveal melanoma is predominantly an adult malignancy, with Ireland reporting one of the highest incidence rates at 9.5 cases per million adults annually. Paediatric uveal melanoma is extremely rare, accounting for fewer than 1% to 2% of all uveal melanoma cases in published series.

**Methods:**

This retrospective study included all patients under 18 years of age diagnosed with uveal melanoma in Ireland between 2010 and 2024. Data were collected from medical records and included demographics, tumour location and dimensions, histopathology or cytology (where available), presence of metastasis, treatment modality, baseline and final visual acuity, intraocular pressure, and survival outcomes.

**Results:**

Six Caucasian patients (five male, one female), aged 6 to 17 years, were identified. Tumour locations included five choroidal and one ciliochoroidal melanoma. Treatment modalities included enucleation (*n* = 2), plaque brachytherapy (two Ruthenium-106, one Iodine-125), and proton beam radiotherapy (*n* = 1). Histopathological analysis was available in two cases, revealing one mixed cell type and one spindle B melanoma. Fine needle aspiration biopsy was performed in two patients. Mean follow-up was 30 months (median 24; range 12–178). All cases remained metastasis-free at last follow-up.

**Conclusions:**

This national case series highlights the rarity and clinical relevance of paediatric uveal melanoma. Outcomes have been favourable, but recent case clustering supports the need for international comparative data collection. Early diagnosis and appropriate treatment help preserve vision and reduce morbidity. Molecular profiling, where possible, may guide metastatic risk assessment. Further studies are needed to confirm international incidence trends.

## Introduction

Uveal melanoma is the most common primary intraocular malignancy in adults, with an estimated overall incidence of 6–7 cases per million per year [1]. It primarily affects older adults, with the average age at diagnosis ranging from 60 to 62 years [2]. Among Caucasian populations, the average annual incidence across all age groups is estimated to be approximately 5 to 8 new cases per million persons per year [3, 4].

Congenital uveal melanoma is exceedingly rare, with only a small number of cases reported in the literature [5]. Paediatric uveal melanoma, affecting children and young adults, is also rare, accounting for fewer than 1% to 2% of all uveal melanoma cases [6–8]. In children, uveal melanoma tends to occur most often during the teenage years, primarily in the choroid, followed by the iris and ciliary body [6]. A population-based study from Finland found that individuals under 25 years of age accounted for 1.3% of uveal melanoma cases, with only 0.6% occurring in those aged 20 or younger [9]. Compared with older adults, younger and middle-aged patients tend to present with smaller basal tumour dimensions and experience lower rates of tumour-related metastasis and mortality [7].

## Methods

This retrospective study included all patients under 18 years of age diagnosed with uveal melanoma at the Ocular Oncology Service, Royal Victoria Eye and Ear Hospital, Dublin, Ireland, between 2010 and 2024. Data collected included demographics, tumour location and size, histology and cytology (where available), presence of metastasis, treatment modality, baseline and final visual acuity (VA), intraocular pressure (IOP), and survival.

## Results

Over the study period, six patients under 18 years of age were diagnosed with uveal melanoma (Table 1). There were five males and one female, aged 6–17 years (mean age 12 ± 3.7). All were of Caucasian ethnicity. Tumour sites included five choroidal and one ciliochoroidal melanoma.

**Table 1:** Summary of clinical features, management, and outcomes.

Case	Year	Age (years)	Sex	Eye	Symptoms	VA at Presentation	IOP at Presentation (mmHg)	Location	Tumour Size (mm)	TNM stage^a^	Histology / Genetics	Treatment	Outcome	Follow-up (months)
**1**	2010	10	F	L	Reduced VA	PL	14	Choroid	14×11×10	pT3a	Spindle B, pT3a, 6p gain, 8q/8p amp, no M3	Enucleation	No recurrence (NR)	178
**2**	2021	12	M	L	Reduced VA	NLP	16	Choroid	9×6×5	pT2a	Mixed cell, no BAP1 loss, no M3/Chr8 abn	Enucleation	NR	51
**3**	2022	13	M	R	Visual distortion	6–6	11	Choroid	14×12.6×4	T2a	No biopsy	Ruthenium-106 plaque	NR, VA 6/6, IOP 11	30
**4**	2024	14	M	R	Reduced VA	6/60	18	Choroid	13.8×13.7×6.2	T3a	FNAB acellular	Iodine-125 plaque + IVT Avastin	NR, VA PH 6/36, IOP 10	18
**5**	2024	17	M	R	Visual distortion	6–6	21	Choroid	5.2×3.9×1.5	T1a	No biopsy	Proton beam	NR. VA 6/6, IOP 19	12
**6**	2024	6	M	R	Reduced VA	6/38	17	Ciliochoroidal	13.3×13.5×5.7	T2	FNAB: malignant melanoma, SOX10 + , BAP1 loss, DNA fail	Ruthenium-106 plaque	NR, VA 6/30, IOP 9	12

*F* female, *M* male, *L* Left eye, *R* Right eye, *VA* visual acuity, *PL* Perception of light, *NLP* No light perception, *N/a* not applicable, *FNAB* fine needle aspiration biopsy, *NR* no recurrence.

^a^AJCC Classification.

Each case was discussed with at least one international oncology centre to support diagnosis and guide management. Treatments included enucleation (*n* = 2), plaque brachytherapy (Ruthenium-106, *n* = 2; Iodine-125, *n* = 1), and proton beam radiotherapy (*n* = 1). Histopathology was available in two cases; fine-needle aspiration biopsy (FNAB) was performed in two, yielding one cytological diagnosis and one acellular sample. No biopsy was undertaken in the remaining two.

Baseline chest X-ray and liver ultrasound were performed in all cases, and all patients were referred for paediatric medical oncology evaluation and follow-up; clinical examination and liver ultrasound every six months for the first five years and annually thereafter. Ocular oncology follow-up visits were scheduled at 2, 4, 8 and 12 months post radiation treatment, then six-monthly to year 3, and annually thereafter. Visits included a comprehensive eye examination with visual acuity, intraocular pressure, biomicroscopy (slit lamp and indirect), optical coherence tomography (OCT), fundus imaging, and ultrasonography. The patients were followed up for an average of 30 months (median 24 months; range 12–178 months). To date, no tumour recurrence or metastatic disease has been identified within the cohort.

A summary of the patients’ year of presentation, age, presenting symptoms, clinical findings and management are presented in Table 1. All patients presented to local optometry/ophthalmology/school vision screening services with either reduced visual acuity or new onset disturbance of vision in the affected eye. Other relevant clinical findings, imaging studies and pathology/cytology findings are outlined here.

### Case 1 (2010)

Slit lamp examination revealed a grey ocular mass arising from the choroid near the optic nerve head, with a total serous retinal detachment. Magnetic resonance imaging (MRI) of the orbits with contrast revealed a left-sided intraocular mass located in the medial aspect of the globe.

**Fig. 1:**
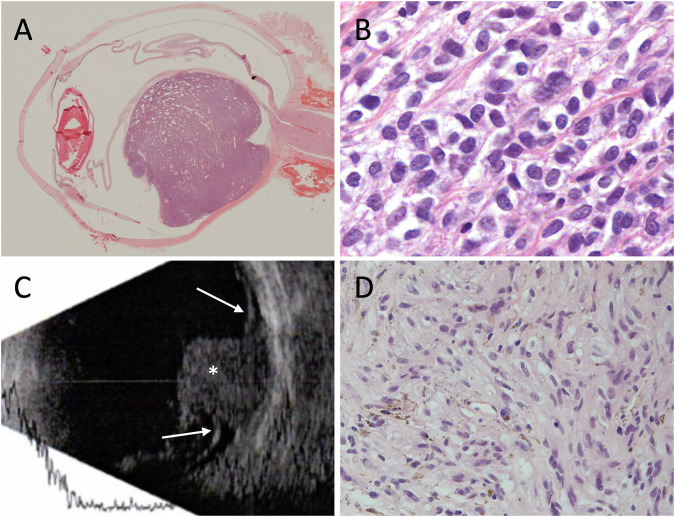
Histopathology and imaging findings in two enucleated eyes with choroidal melanoma. Case 1: **A** low-power view of the enucleation specimen shows a large, non-pigmented choroidal tumour (haematoxylin and eosin [H&E], 1× magnification); **B** high-power view reveals sheets of plump, ovoid tumour cells with coarse chromatin and abundant cytoplasm (H&E, 60× magnification). Case 2: **C** B-scan ultrasonography of the left eye demonstrates a hyper-reflective intraocular mass (asterisk) with associated retinal detachment (arrows); **D** enucleation specimen shows mixed cell-type melanoma, highlighting an area with spindle cells with distinct cell membranes, hyperchromatic nuclei and inconspicuous nucleoli (H&E, 40× magnification).

The patient underwent enucleation of the left eye with insertion of a mesh-wrapped bioceramic orbital implant (FCI Ophthalmics Inc., Massachusetts, USA). Histological examination demonstrated a mushroom-shaped amelanotic melanoma, measuring 14 × 11 × 10 mm, with spindle cell type-B morphology, along with invasion into the sclera and retina. Extensive intrascleral vascular invasion and breach of Bruch’s membrane were present. There was no mitosis, necrosis, or extraocular extension. The tumour was staged as pT3a (Fig. 1A, B). Tumour cells showed diffuse positivity for Melan-A, and BRCA1-associated protein 1 (BAP1) expression was retained. Fluorescence in situ hybridisation (FISH) analysis revealed full or partial trisomy of chromosome 8, with no chromosome 3 abnormalities. Whole genome copy number analysis confirmed chromosome 8 amplification and no evidence of monosomy 3.

### Case 2 (2021)

Slit lamp examination revealed a pedunculated, creamy-white mass with overlying intact retinal vessels, associated exudate, haemorrhage, and an exudative retinal detachment. B-scan ultrasonography demonstrated an elevated, choroidal lesion with internal vascular flow and associated exudative retinal detachment (Fig. 1C).

The patient underwent enucleation of the left eye with insertion of a Medpor® orbital implant (Stryker, Michigan, USA). Histological examination revealed a pigmented choroidal mass measuring 9 × 6 × 5 mm, composed of mixed cell-type melanoma, with >10% large epithelioid cells and <90% spindle cells (Fig. 1D). There was a breach of Bruch’s membrane. No evidence of mitosis, necrosis, or extraocular extension was observed. The pathological stage was pT2a. Ki-67 showed no evidence of proliferative activity. BAP1 immunohistochemistry demonstrated intact nuclear expression in both tumour and non-tumour cells. FISH analysis revealed no evidence of monosomy 3 or chromosome 8 abnormalities.

### Case 3 (2022)

**Fig. 2:**
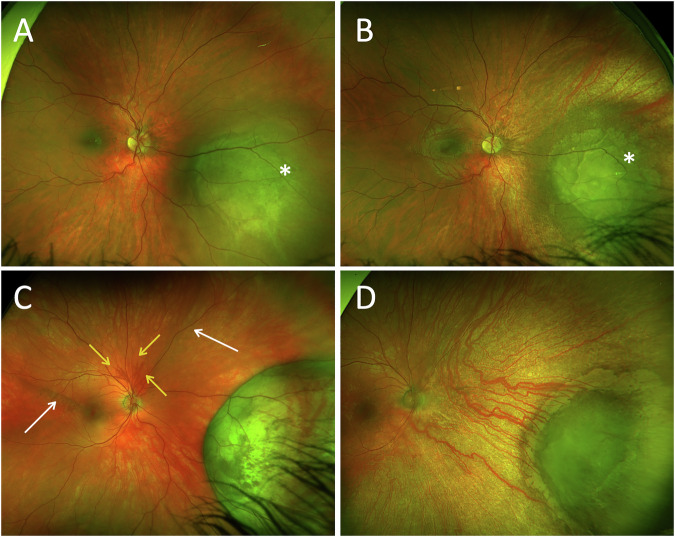
Fundus imaging before and after plaque brachytherapy in two cases of choroidal melanoma. Case 3: **A** baseline fundus image demonstrates a choroidal mass (asterisk) prior to treatment; **B** follow-up fundus image at 30 months post–Ruthenium-106 plaque brachytherapy shows a marked reduction in lesion size. Case 4: **C** baseline fundus image shows a grey choroidal mass with optic disc neovascularisation (yellow arrows) and chronic retinal detachment (white arrows); **D** one year post-treatment with Iodine-125 plaque brachytherapy and intravitreal Avastin®, follow-up imaging shows regression of neovascularisation and reduced tumour size.

Examination revealed a grey choroidal mass in the inferonasal quadrant (Fig. 2A). Fundus fluorescein angiography (FFA) showed irregular hyperfluorescence in the early phase. Indocyanine green angiography (ICG) demonstrated an area of hypocyanescence without abnormal vascular pattern or late leakage. B-scan ultrasonography revealed an inferonasally located, elevated choroidal-based lesion measuring 14 × 12.6 × 4 mm, with internal vascular flow.

The patient underwent Ruthenium-106 plaque brachytherapy to the right eye. The radioactive plaque delivered a dose of 88.6 Grey to a depth of 4.0 mm over 112 h. No biopsy was performed.

At three months post-treatment, VA was 6/7.5 and IOP was 32 mmHg, which was successfully managed with twice-daily Cosopt® (dorzolamide/timolol). At five months, B-scan ultrasonography demonstrated a significant reduction in tumour size to 8.1 × 7.6 × 1.0 mm, with no internal vascular flow. At 30-month follow-up (Fig. 2B), VA had improved to 6/6, and IOP remained within normal limits without topical medication.

### Case 4 (2024)

Fundus examination revealed a grey choroidal mass with associated chronic exudative retinal detachment and neovascularisation of the optic disc (Fig. 2C), confirmed on B-scan ultrasonography as an inferonasal choroidal-based tumour measuring 13.8 × 13.7 × 6.2 mm. Macular OCT showed a pocket of subretinal fluid. MRI of the orbits revealed a sessile intraocular mass in the right eye, measuring 10 mm in diameter, with intrinsic T1 hyperintensity.

The patient underwent Iodine-125 plaque brachytherapy to the right eye. The radioactive plaque delivered a dose of 83.65 Grey to a depth of 6.20 mm over 119 h. A transscleral 30 G FNAB was attempted during plaque placement but yielded an acellular sample. Intravitreal Avastin® (bevacizumab) was administered at the time of plaque removal, followed by two additional injections at four-week intervals.

At three months post-treatment, VA had declined to counting fingers (CF), and IOP was 19 mmHg. The lesion had decreased in size, and the neovascularisation had completely regressed. At the 18-month follow-up, best corrected visual acuity (BCVA) had improved to 6/36, IOP was 10 mmHg, and the lesion had further regressed, with no recurrence of optic disc neovascularisation (Fig. 2D) and complete resolution of subretinal fluid.

### Case 5 (2024)

**Fig. 3:**
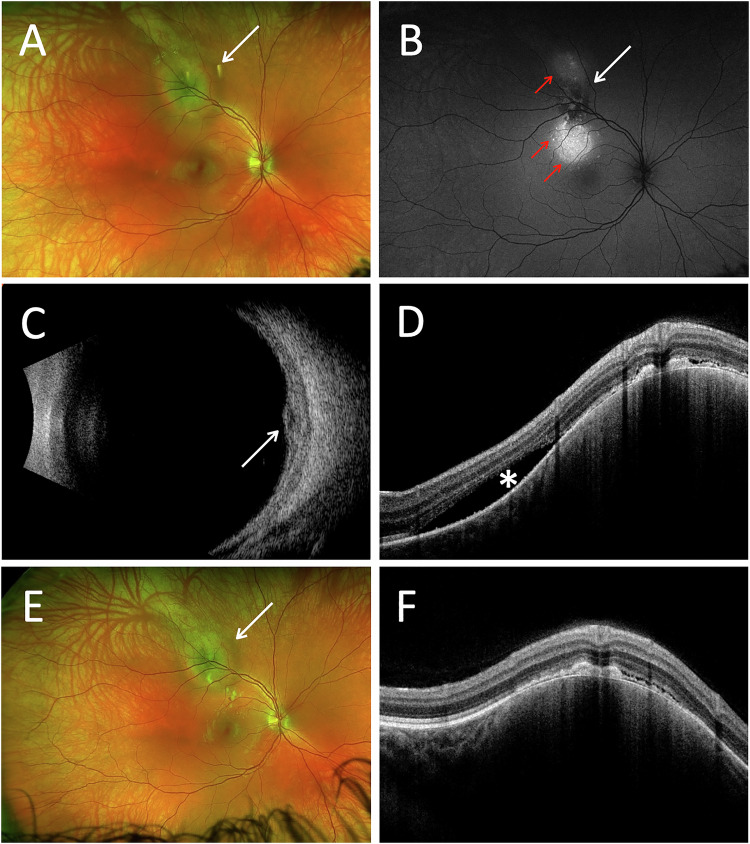
Multimodal imaging of a small choroidal melanoma before and after proton beam therapy. Case 5: Baseline fundus image **A**, FAF **B**, and B-scan ultrasound **C** demonstrate a choroidal mass (white arrow). Note the hyperautofluorescent areas of RPE changes (red arrows) on FAF, corresponding to subretinal fluid seen on baseline OCT (**D**). Follow-up fundus image (**E**) and OCT (**F**) 10 months after proton beam therapy show resolution of subretinal fluid following treatment.

Fundus examination revealed a yellow-green choroidal mass at the superior arcade (Fig. 3A, B), with associated subretinal fluid (SRF) over the lesion on OCT (Fig. 3D). FFA showed early hyperfluorescence, and ICG revealed an area of hypocyanescence with a peripheral ring of hypercyanescence. B-scan ultrasonography demonstrated a small, elevated, hypoechoic choroidal lesion measuring 1.4 mm in thickness (Fig. 3C).

The patient underwent proton beam radiotherapy at Clatterbridge Cancer Centre, Liverpool, United Kingdom. No biopsy was performed.

At the most recent follow-up, 12 months post-treatment, VA remained 6/6 and IOP was 19 mmHg. Fundus imaging demonstrated a reduction in lesion size (Fig. 3E), confirmed on B-scan. OCT revealed only trace residual subretinal fluid (Fig. 3F).

### Case 6 (2024)

**Fig. 4:**
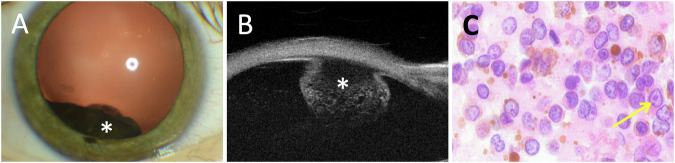
Clinical imaging and cytologic confirmation of pigmented ciliochoroidal melanoma. Case 6: Anterior segment photograph of the right eye **A** shows a pigmented ciliochoroidal lesion visible through the pupil (asterisk). The inferotemporal ciliochoroidal lesion is visible on ultrasound biomicroscopy (**B**). Cytology cell block (**C**) reveals large, atypical cells with irregular cell membranes and prominent nucleoli (yellow arrow) (H&E, 60× magnification).

Slit lamp examination revealed a darkly pigmented, ciliochoroidal lesion visible through the pupil (Fig. 4A). Fundus examination identified a protruding mass in the inferotemporal quadrant. Anterior segment fluorescein angiography demonstrated subtle leakage of fluorescein.

MRI of the orbits showed a right ciliochoroidal lesion measuring 0.7 × 0.62 × 0.68 cm (estimated volume ~0.15 mL). Ultrasound biomicroscopy (Fig. 4B) and B-scan ultrasonography confirmed a lesion extending from 6 to 9 o’clock, with an anteroposterior diameter of 13.3 mm, circumferential extension of 13.5 mm, and thickness of 5.7 mm. Cytology after 30 G FNAB revealed a cellular sample containing pigmented large epithelioid cells with irregular membranes and prominent nucleoli (Fig. 4C). The atypical cells were positive for SOX10 immunostaining supporting the diagnosis of malignant melanoma. BAP1 immunohistochemistry demonstrated loss of nuclear BAP1 expression in tumour cells.

The patient underwent Ruthenium-106 plaque brachytherapy to the right eye using a 17.8 mm plaque that delivered a dose of 80.30 Grey to a depth of 5.70 mm over a period of 67 h. At three months post-operatively, the lesion had reduced in size. At the latest follow-up at 12 months BCVA was 6/30 and IOP was 9 mmHg.

## Discussion

Uveal melanoma is the most common primary intraocular malignancy in adults, with an estimated overall incidence of 6–7 cases per million per year in Europe and North America [1], and a mean age at diagnosis of 60–62 years [2]. Among Caucasian populations, the average annual incidence across all age groups is estimated to be approximately 5 to 8 new cases per million persons per year [3, 4, 7]. In Ireland, and Scandinavia the incidence of uveal melanoma has been reported to be higher than in other European and global studies, with a greater prevalence among males (11.1 per million in males vs 8.2 per million in females) [10]. Paediatric uveal melanoma, affecting children and young adults (variably defined as under 21 to 25 years of age in different studies), is extremely rare, accounting for fewer than 1–2% of all uveal melanoma cases [6–9].

This series reports the clinical characteristics, treatment, and outcomes of paediatric uveal melanoma cases seen at the Irish Ocular Oncology Service, Royal Victoria Eye and Ear Hospital, Dublin, since 2010, when ocular oncology services in the Republic of Ireland were centralised to a single centre. Although determining the precise incidence of paediatric uveal melanoma in a small population is challenging, extrapolation from international data [4, 6–8] suggests that Ireland would be expected to encounter approximately one paediatric case every two to five years. Our findings broadly align with this estimate; however, the temporal distribution is noteworthy. Six paediatric cases were identified between 2010 and 2025, with five diagnosed in the past four years: one in 2021, one in 2022, and three in 2024. Previous studies have reported a slight female predominance in paediatric uveal melanoma [4, 6]. In contrast, five of the six cases in our series occurred in males, suggesting a potentially divergent trend that may warrant further investigation, although the numbers are too small to draw definitive conclusions.

The most common presenting symptom in adults with primary uveal melanoma is blurred vision, although approximately one-third of patients are asymptomatic at the time of diagnosis [11, 12]. Other frequently reported symptoms include photopsia, floaters, visual field defects, and, less commonly, pain or metamorphopsia [11, 12]. In our series, two patients presented with reduced vision as the initial symptom. One patient reported signs consistent with retinal detachment, while another underwent MRI to investigate optic disc pallor, which led to an incidental diagnosis of uveal melanoma. Incidental detection of uveal melanoma on MRI has been described in the literature; one study reported three asymptomatic adult patients in whom melanoma was identified only through imaging [13]. In another case from our series, the tumour was detected incidentally during a routine optometry examination. One study showed that in the UK optometrists initiate the referral process in approximately 68% of adult uveal melanoma diagnoses [11]. One patient in this series was referred after a failed school vision screening, despite having no self-reported symptoms.

Treatment options for uveal melanoma do not differ significantly between adults and children [14]. Enucleation is typically reserved for larger tumours or those in locations unsuitable for radiotherapy [15]. Radiation-based treatments, including plaque brachytherapy and proton beam radiotherapy, are often preferred over surgical resection due to their ability to precisely target the tumour while preserving surrounding ocular structures [16]. These modalities offer both eye-sparing and sight-preserving benefits [15]. In our series, two patients with a pre-treatment BCVA of 6/6 maintained this level of vision following plaque brachytherapy and proton beam radiotherapy, respectively. Two additional patients experienced minor improvement in VA after brachytherapy. Both enucleation and radiation therapy have shown comparable survival outcomes, with no clear advantage in terms of metastatic risk. However, tumour thickness can limit the use of radiotherapy, as lesions thicker than 6 mm are associated with a higher risk of treatment-related complications [16].

Survival outcomes in paediatric uveal melanoma are generally more favourable than in adult cases. Children under 18 years have been shown to have better survival than young adults aged 18 to 24 years, with TNM stage identified as a strong predictor of prognosis [9, 17]. Ciliary body involvement and higher TNM stage were both associated with increased mortality among children under 18 years of age [5, 17]. Furthermore, the presence of extraocular extension was linked to poor prognosis and worse survival [5]. Male sex is associated with improved survival, whereas congenital melanocytosis significantly increases mortality risk, with a 5.6-fold higher likelihood of metastasis in affected patients [17].

Previous studies have shown that aggressive tumour cell type is associated with poor prognosis and worse outcome in uveal melanoma [18, 19]. In adults cell morphology has been shown to have important prognostic implications in choroidal and ciliary body melanoma, although this is not the case in iris melanoma [19, 20]. Spindle cell uveal melanomas, composed of more than 90 percent spindle cells, are associated with the most favourable outcomes. Epithelioid cell melanomas, in which more than 90 percent of the cells are epithelioid, have the poorest prognosis. Mixed cell melanomas, which contain fewer than 90% spindle cells and more than 10% epithelioid cells, carry an intermediate prognosis [19, 20]. Al-Jamal RT, et al. reported that cell type was not significantly associated with survival in paediatric population [17].

In adults, patients with uveal melanoma have an estimated 50% risk of metastasis [21, 22]. While tumour size and depth are key prognostic factors in uveal melanoma, molecular biomarkers also play a critical role in predicting outcomes [15]. Monosomy 3 and alterations in chromosome 8, including gain or loss in either arm, are associated with higher risk of metastasis and mortality [20, 23, 24], while loss of BAP1 expression correlates with poorer disease-specific survival [18]. Loss of nuclear BAP1 expression by immunohistochemistry serves as a surrogate marker for pathogenic BAP1 mutations [5, 18]. Conversely, gain of chromosome 6p is linked to a more favourable prognosis [20, 25, 26].

In paediatric uveal melanoma, tumours tend to be smaller, located further from the macula and optic disc, and less often show extraocular extension or metastasis [7]. In this context, molecular profiling is particularly useful to stratify risk and guide follow-up. Among the six patients in our cohort tissue was available in three (two enucleations and 1 FNAB; 1 acellular FNAB). One patient demonstrated chromosome 8 gain on FISH analysis, with additional amplification of chromosomes 8q and 8p identified on molecular genetic testing. Another patient, treated with Ruthenium-106 brachytherapy, demonstrated loss of BAP1.

Germline BRCA1-associated protein 1 tumour predisposition syndrome (BAP1-TPDS) is associated with an increased risk of developing uveal melanoma, cutaneous melanoma, renal cell carcinoma, and mesothelioma [27–29]. Although BAP1 germline pathogenic variants are identified in only 2–5% of patients with uveal melanoma [28, 30] and in about twenty percent in familial uveal melanoma [28, 31], uveal melanoma is the most frequent tumour observed in BAP1-TPDS [28, 29]. When present, it often exhibits distinct phenotypes, including bilateral tumours, earlier age of onset, and a familial inheritance pattern [28]. Germline pathogenic variants in BRCA2 [32] and MBD4 (Methyl-CpG-binding domain protein 4) [33] have also been reported. Biallelic MBD4 loss due to monosomy 3 leads to hypermutated uveal melanoma tumours which may respond to immune checkpoint inhibitors, unlike classic uveal melanoma [34, 35]. In our own overall uveal melanoma patient database definitive cancer predisposition syndromes occur infrequently. Notably, none of the patients in this paediatric cohort were diagnosed with a genetic syndrome or had a significant family history of cancer. Neither did any of our patient group exhibit ocular or oculodermal melanocytosis.

This national case series highlights the rarity but clinical significance of paediatric uveal melanoma. Although outcomes have been favourable, the recent clustering of cases warrants continued monitoring and comparison with data from other international ocular oncology centres. Early diagnosis and carefully selected treatment can help preserve vision and limit morbidity. Where possible, molecular profiling should be pursued to support long-term-metastatic-risk assessment. Further collaborative studies are needed to validate current incidence rates of uveal melanoma in the paediatric population and optimise management strategies for this rare patient group.

## Summary

### What was known before:

- Uveal melanoma is a rare cancer. Paediatric uveal melanoma is extremely rare, accounting for fewer than 1% to 2% of all uveal melanoma cases.

### What this study adds:

- In a national ocular oncology centre one case of paediatric uveal melanoma was diagnosed over the time span 2010–2020 with 5 paediatric uveal melanoma cases diagnosed between 2021 and 2024.
- Review of comparative data from other centres over a similar time span would help verify if this represents a chance cluster or a real trend in incidence rates.

## Data Availability

All data analysed in this study are included in this published article.

## References

[1] Spaeth EB. Ocular tumors: a study of incidence of the various types and their mortality rates. AMA Arch Ophthalmol. 1951;46:421–3. 10.1001/archopht.1951.01700020432007. 14868062

[2] Andreoli MT, Mieler WF, Leiderman YI. Epidemiological trends in uveal melanoma. Br J Ophthalmol. 2015;99:1550–3. 10.1136/bjophthalmol-2015-306810. 25904122 10.1136/bjophthalmol-2015-306810

[3] Singh AD, Turell ME, Topham AK. Uveal melanoma: trends in incidence, treatment, and survival. Ophthalmology. 2011;118:1881–5. 10.1016/j.ophtha.2011.01.040. 21704381 10.1016/j.ophtha.2011.01.040

[4] Fry MV, Augsburger JJ, Hall J, Corrêa ZM. Posterior uveal melanoma in adolescents and children: current perspectives. Clin Ophthalmol. 2018;12:2205–12. 10.2147/OPTH.S142984. 30464381 10.2147/OPTH.S142984 PMC6228084

[5] van Poppelen NM, Cassoux N, Turunen JA, Naus NC, Verdijk RM, Vaarwater J, et al. The pediatric and young adult choroidal and ciliary body melanoma genetic study, a survey by the European ophthalmic oncology group. Investig Ophthalmol Vis Sci. 2024;65:12 10.1167/iovs.65.4.12. 10.1167/iovs.65.4.12 PMC10996971 38573618

[6] Shields CL, Kaliki S, Arepalli S, Atalay HT, Manjandavida FP, Pieretti G, et al. Uveal melanoma in children and teenagers. Saudi J Ophthalmol. 2013;27:197–201. 10.1016/j.sjopt.2013.06.013. 24227986 10.1016/j.sjopt.2013.06.013 PMC3770213

[7] Shields CL, Kaliki S, Furuta M, Mashayekhi A, Shields JA. Clinical spectrum and prognosis of uveal melanoma based on age at presentation in 8,033 cases. Retina. 2012;32:1363–72. 10.1097/IAE.0b013e31824d09a8. 22466491 10.1097/IAE.0b013e31824d09a8

[8] Liu YM, Li Y, Wei WB, Xu X, Jonas JB. Clinical characteristics of 582 patients with uveal melanoma in China. PLoS ONE. 2015;10:e0144562 10.1371/journal.pone.0144562. 26645696 10.1371/journal.pone.0144562 PMC4672905

[9] Al-Jamal RT, Kivelä T. Uveal melanoma among Finnish children and young adults. J AAPOS. 2014;18:61–6. 10.1016/j.jaapos.2013.11.006. 24568985 10.1016/j.jaapos.2013.11.006

[10] Baily C, O’Neill V, Dunne M, Cunningham M, Gullo G, Kennedy S, et al. Uveal melanoma in Ireland. Ocul Oncol Pathol. 2019;5:195–204. 10.1159/000492391. 31049328 10.1159/000492391 PMC6489068

[11] Damato EM, Damato BE. Detection and time to treatment of uveal melanoma in the United Kingdom: an evaluation of 2384 patients. Ophthalmology. 2012;119:1582–9. 10.1016/j.ophtha.2012.01.048. 22503229 10.1016/j.ophtha.2012.01.048

[12] Krantz BA, Dave N, Komatsubara KM, Marr BP, Carvajal RD. Uveal melanoma: epidemiology, etiology, and treatment of primary disease. Clin Ophthalmol. 2017;11:279–89. 10.2147/OPTH.S89591. 28203054 10.2147/OPTH.S89591 PMC5298817

[13] Tsukikawa M, Akinpelu B, Wangaryattawanich P, Scherpelz K, Stacey AW. Uveal melanoma incidentally diagnosed with neuroimaging, a case series of 3 patients. Radiol Case Rep. 2021;17:54–9. 10.1016/j.radcr.2021.09.064. 34765060 10.1016/j.radcr.2021.09.064 PMC8572855

[14] PDQ Pediatric Treatment Editorial Board. Childhood Intraocular (Uveal) Melanoma Treatment (PDQ®): Health Professional Version. PDQ Cancer Information Summaries [Internet]. 2024. National Cancer Institute (US). Available from: https://www.ncbi.nlm.nih.gov/books/NBK390611/. 31909938

[15] Hanratty K, Finegan G, Rochfort KD, Kennedy S. Current Treatment of Uveal Melanoma. Cancers. 2025;17:1403 10.3390/cancers17091403. 40361330 10.3390/cancers17091403 PMC12071000

[16] Kulbay M, Marcotte E, Remtulla R, Lau THA, Paez-Escamilla M, Wu KY, et al. Uveal melanoma: comprehensive review of its pathophysiology, diagnosis, treatment, and future perspectives. Biomedicines. 2024;12:1758 10.3390/biomedicines12081758. 39200222 10.3390/biomedicines12081758 PMC11352094

[17] Al-Jamal RT, Cassoux N, Desjardins L, Damato B, Konstantinidis L, Coupland SE, et al. The pediatric choroidal and ciliary body melanoma study: a survey by the european ophthalmic oncology group. Ophthalmology. 2016;123:898–907. 10.1016/j.ophtha.2015.12.024. 26854035 10.1016/j.ophtha.2015.12.024

[18] Kennedy S, Owens S, Ivers L, Hegarty C, O’Neill V, Berenguer-Pina JJ, et al. Prognostic value of BAP1 protein expression in uveal melanoma. Am J Surg Pathol. 2024;48:329–36. 10.1097/PAS.0000000000002176. 38238977 10.1097/PAS.0000000000002176 PMC10876168

[19] Lamas NJ, Martel A, Nahon-Estève S, Goffinet S, Macocco A, Bertolotto C, et al. Prognostic biomarkers in uveal melanoma: the status Quo, recent advances and future directions. Cancers. 2021;14:96 10.3390/cancers14010096. 35008260 10.3390/cancers14010096 PMC8749988

[20] Kaliki S, Shields CL, Shields JA. Uveal melanoma: estimating prognosis. Indian J Ophthalmol. 2015;63:93–102. 10.4103/0301-4738.154367. 25827538 10.4103/0301-4738.154367 PMC4399142

[21] Tura A, Merz H, Reinsberg M, Lüke M, Jager MJ, Grisanti S, et al. Analysis of monosomy-3 in immunomagnetically isolated circulating melanoma cells in uveal melanoma patients. Pigment Cell Melanoma Res. 2016;29:583–9. 10.1111/pcmr.12507. 27390171 10.1111/pcmr.12507

[22] Seedor RS, Orloff M, Sato T. Genetic landscape and emerging therapies in uveal melanoma. Cancers. 2021;13:5503 10.3390/cancers13215503. 34771666 10.3390/cancers13215503 PMC8582814

[23] Prescher G, Bornfeld N, Hirche H, Horsthemke B, Jöckel KH, Becher R. Prognostic implications of monosomy 3 in uveal melanoma. Lancet. 1996;347:1222–5. 10.1016/s0140-6736(96)90736-9. 8622452 10.1016/s0140-6736(96)90736-9

[24] Ewens KG, Kanetsky PA, Richards-Yutz J, Al-Dahmash S, De Luca MC, Bianciotto CG, et al. Genomic profile of 320 uveal melanoma cases: chromosome 8p-loss and metastatic outcome. Investig Ophthalmol Vis Sci. 2013;54:5721–9. 10.1167/iovs.13-12195. 23821189 10.1167/iovs.13-12195

[25] Singh M, Durairaj P, Yeung J. Uveal melanoma: a review of the literature. Oncol Ther. 2018;6:87–104. 10.1007/s40487-018-0056-8. 32700136 10.1007/s40487-018-0056-8 PMC7359963

[26] Damato B, Dopierala JA, Coupland SE. Genotypic profiling of 452 choroidal melanomas with multiplex ligation-dependent probe amplification. Clin Cancer Res. 2010;16:6083–92. 10.1158/1078-0432.CCR-10-2076. 20975103 10.1158/1078-0432.CCR-10-2076

[27] Pilarski R, Byrne L, Carlo MI, Hanson H, Cebulla C, Abdel-Rahman M. BAP1 tumor predisposition syndrome. 2016 Oct 13 [updated 2024 Dec 5]. In: Adam MP, Feldman J, Mirzaa GM, Pagon RA, Wallace SE, Amemiya A, editors. GeneReviews® [Internet]. Seattle (WA): University of Washington, Seattle; 1993–2025.27748099. 27748099

[28] Singh N, Singh R, Bowen RC, Abdel-Rahman MH, Singh AD. Uveal Melanoma in BAP1 tumor predisposition syndrome: estimation of risk. Am J Ophthalmol. 2021;224:172–7. 10.1016/j.ajo.2020.12.005. 33316260 10.1016/j.ajo.2020.12.005 PMC8059106

[29] Masoomian B, Shields JA, Shields CL. Overview of BAP1 cancer predisposition syndrome and the relationship to uveal melanoma. J Curr Ophthalmol. 2018;30:102–9. 10.1016/j.joco.2018.02.005. 29988936 10.1016/j.joco.2018.02.005 PMC6034168

[30] Repo P, Järvinen RS, Jäntti JE, Markkinen S, Täll M, Raivio V, et al. Population-based analysis of BAP1 germline variations in patients with uveal melanoma. Hum Mol Genet. 2019;28:2415–26. 10.1093/hmg/ddz076. 31058963 10.1093/hmg/ddz076

[31] Rai K, Pilarski R, Boru G, Rehman M, Saqr AH, Massengill JB, et al. Germline BAP1 alterations in familial uveal melanoma. Genes Chromosomes Cancer. 2017;56:168–74. 10.1002/gcc.22424. 27718540 10.1002/gcc.22424 PMC5490375

[32] Johansson PA, Nathan V, Bourke LM, Palmer JM, Zhang T, Symmons J, et al. Evaluation of the contribution of germline variants in BRCA1 and BRCA2 to uveal and cutaneous melanoma. Melanoma Res. 2019;29:483–90. 10.1097/CMR.0000000000000613. 31464824 10.1097/CMR.0000000000000613 PMC6716616

[33] Villy MC, Le Ven A, Le Mentec M, Masliah-Planchon J, Houy A, Bièche I, et al. Familial uveal melanoma and other tumors in 25 families with monoallelic germline MBD4 variants. J Natl Cancer Inst. 2024;116:580–7. 10.1093/jnci/djad248. 38060262 10.1093/jnci/djad248

[34] Saint-Ghislain M, Derrien AC, Geoffrois L, Gastaud L, Lesimple T, Negrier S, et al. MBD4 deficiency is predictive of response to immune checkpoint inhibitors in metastatic uveal melanoma patients. Eur J Cancer. 2022;173:105–12. 10.1016/j.ejca.2022.06.033. 35863105 10.1016/j.ejca.2022.06.033

[35] Derrien AC, Rodrigues M, Eeckhoutte A, Dayot S, Houy A, Mobuchon L, et al. Germline MBD4 mutations and predisposition to uveal melanoma. J Natl Cancer Inst. 2021;113:80–7. 10.1093/jnci/djaa047. 32239153 10.1093/jnci/djaa047 PMC7781447

